# High Radiation Resistance in the Binary W‐Ta System Through Small V Additions: A New Paradigm for Nuclear Fusion Materials

**DOI:** 10.1002/advs.202417659

**Published:** 2025-03-07

**Authors:** Matheus A. Tunes, Darren Parkison, Bochuan Sun, Patrick Willenshofer, Sebastian Samberger, Christoph Frühwirth, Shalini Tripathi, Benjamin K. Derby, Jon Kevin S. Baldwin, Saryu J. Fensin, Damian Sobieraj, Jan S. Wróbel, Jesper Byggmästar, Stefan Pogatscher, Enrique Martinez, Duc Nguyen‐Manh, Osman El‐Atwani

**Affiliations:** ^1^ Department of Metallurgy Chair of Non‐Ferrous Metallurgy Montanuniversität Leoben Leoben Steiermark 8700 Austria; ^2^ Materials Science and Technology Division Los Alamos National Laboratory Los Alamos New Mexico 87545 USA; ^3^ Department of Nuclear Engineering University of California at Berkeley Berkeley California 94720 USA; ^4^ Departments of Mechanical Engineering and Materials Science and Engineering Clemson University Clemson South Carolina 29634 USA; ^5^ Reactor Materials Group, Nuclear Sciences Division Pacific Northwest National Laboratory Richland Washington 99352 USA; ^6^ Center for Integrated Nanotechnologies Los Alamos National Laboratory Los Alamos New Mexico 87545 USA; ^7^ Faculty of Materials Science and Engineering Warsaw University of Technology Warsaw 02‐507 Poland; ^8^ Department of Physics University of Helsinki Helsinki 00014 Finland; ^9^ Materials Division, United Kingdom Atomic Energy Authority Culham Campus Abingdon OX14 3DB UK; ^10^ Department of Materials University of Oxford Parks Road Oxford OX1 3PH UK

**Keywords:** fusion materials, high entropy alloys, refractory high entropy alloys, radiation damage, thermonuclear fusion, in situ TEM

## Abstract

Refractory High‐Entropy Alloys (RHEAs) are promising candidates for structural materials in nuclear fusion reactors, where W‐based alloys are currently leading. Fusion materials must withstand extreme conditions, including i) severe radiation damage from energetic neutrons, ii) embrittlement due to H and He ion implantation, and iii) exposure to high temperatures and thermal gradients. Recent RHEAs, such as WTaCrV and WTaCrVHf, have shown superior radiation tolerance and microstructural stability compared to pure W, but their multi‐element compositions complicate bulk fabrication and limit practical use. In this study, it is demonstrated that reducing alloying elements in RHEAs is feasible without compromising radiation tolerance. Herein, two Highly Concentrated Refractory Alloys (HCRAs) − W_53_Ta_44_V_3_ and W_53_Ta_42_V_5_ (at.%) − were synthesized and investigated. We found that small V additions significantly influence the radiation response of the binary W–Ta system. Experimental results, supported by *ab‐initio* Monte Carlo simulations and machine‐learning‐driven molecular dynamics, reveal that minor variations in V content enhance Ta–V chemical short‐range order (CSRO), improving radiation resistance in the W_53_Ta_42_V_5_ HCRA. By focusing on reducing chemical complexity and the number of alloying elements, the conventional high‐entropy alloy paradigm is challenged, suggesting a new approach to designing simplified multi‐component alloys with refractory properties for thermonuclear fusion applications.

## Graphical abstract

Refractory high‐entropy alloys exhibit superior radiation tolerance, but face fabrication challenges due to complex compositions. It is demonstrated that reducing the number of alloying elements does not compromise radiation resistance in a novel W‐Ta‐V highly‐concentrated refractory alloy. Experiments and simulations reveal that V‐induced chemical short‐range order enhances alloy stability, offering a pathway to chemically simplified refractory alloys for nuclear fusion applications.

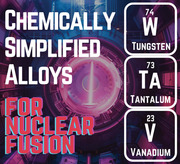

## 1 Introduction

Since the 1970s, materials selection for application in future thermonuclear fusion reactors has posed a significant challenge for materials science and metallurgy.^[^ ^1^, ^2^, ^3^, ^4^, ^5^, ^6^ ^]^ The deuterium‐tritium fusion reaction exposes fusion reactors and their structural materials to exceptionally harsh environmental conditions. To date, considering the design and selection of materials for experimental fusion reactors, the following key factors must be addressed, not exhaustively:^[^ ^1^, ^2^, ^3^, ^4^, ^5^, ^6^ ^]^

- Impact of highly‐energetic fusion neutrons (≈14.1 MeV) resulting in radiation damage;
- Implantation of helium (He) and hydrogen (H) ions at moderate‐to‐low energies, resulting in synergistic radiation damage, inert gas bubbles nucleation, and growth and materials embrittlement; and
- The presence of a plasma with high‐power density, resulting in high‐temperature exposure and thermal gradients.

As the 21st century began, two critical objectives arose that must be addressed to render fusion reactors feasible: i) better controlling the high‐power plasma with ii) concomitant enhancement of its performance.^[^ ^4^ ^]^ For these reasons, materials science has redirected its attention toward better understanding plasma‐materials interactions.^[^ ^7^ ^]^ The surface of plasma‐facing materials will be constantly subjected to the severe degradation forces aforementioned, which can significantly hinder the fusion reactor operation.^[^ ^4^ ^]^ Currently, beryllium (Be),^[^ ^4^, ^8^ ^]^ and tungsten (W)^[^ ^4^ ^]^ are being considered for use in the first‐wall armor, while W is the choice for divertor applications.^[^ ^9^, ^10^, ^11^, ^12^, ^13^, ^14^ ^]^

A significant challenge associated with the potential use of W in fusion reactors was highlighted in a 1998 article by Davis et al.,^[^ ^9^ ^]^ which assessed W for application in the International Thermonuclear Experimental Reactor (ITER). The conclusion of this assessment stated that W was not suitable for use in ITER due to its low strength, limited thermal shock resistance, and high ductile‐brittle transition temperature (DBTT).^[^ ^9^ ^]^ Moreover, beyond the issues of low strength and brittleness, studies on energetic particle irradiation response have revealed a series of irradiation effects and microstructural changes in W that cannot be ignored.^[^ ^15^, ^16^, ^17^ ^]^ These irradiation effects, when emulated with particle accelerators – as recently reviewed and summarized by Harrison^[^ ^17^ ^]^ – include a high density of dislocation loops and the formation of He bubbles,^[^ ^18^, ^19^ ^]^ both of which can further increase W brittleness through irradiation‐induced hardening. Ferroni et al. also reported that when radiation damage is formed in W, even high temperature isochronal annealing is not able to fully recover defects in the W microstructure.^[^ ^20^ ^]^

The challenge associated with improving mechanical properties of W has attracted increased attention of the greater materials and metallurgical engineering communities in the recent years.^[^ ^14^, ^21^ ^]^ Various approaches have been explored, including reducing the grain size of W through methods such as cold‐rolling,^[^ ^21^, ^22^, ^23^, ^24^ ^]^ wire drawing,^[^ ^25^, ^26^, ^27^, ^28^ ^]^ and severe‐plastic deformation (SPD).^[^ ^29^, ^30^ ^]^ Another avenue involves grain‐boundary doping,^[^ ^14^, ^31^, ^32^, ^33^ ^]^ although challenges associated with intragranular fracture and fracture resistance of nanocrystalline W are still pending resolution and can be considered topics for further research.^[^ ^34^, ^35^, ^36^, ^37^ ^]^ Nevertheless, it is worth noting that recent radiation damage studies – carried out with light‐ and heavy‐ion irradiation within in situ Transmission Electron Microscopy (TEM) independently at both the IVEM facility in USA^[^ ^38^ ^]^ and the MIAMI facility in UK^[^ ^39^, ^40^ ^]^ – have shown that even nanocrystalline W experience severe damage from energetic particle irradiation,^[^ ^41^, ^42^, ^43^, ^44^ ^]^ thus raising questions regarding the overall feasibility of proposing W for fusion applications. It is important emphasizing that in situ TEM ion irradiation studies on coarse‐grained W and select W‐alloys also indicated extensive formation of radiation damage defects in a similar manner.^[^ ^45^, ^46^, ^47^ ^]^ Neither an irradiation nor thermal stability study have yet been conducted on doped nanocrystalline W, indicating potential for future research still considering this element a candidate as fusion material.

An alternative to W in fusion applications is presented by the field of high‐entropy alloys (HEAs),^[^ ^48^, ^49^, ^50^, ^51^, ^52^ ^]^ and more specifically due to their high‐temperature stability, the RHEAs.^[^ ^53^, ^54^, ^55^, ^56^ ^]^ Composed of four or more alloying elements in near‐equimolar concentrations, these alloys are designed to maximize configurational entropy and minimize Gibbs free energy, thereby enhancing thermodynamic stability of the solid solution phase. Nanocrystalline RHEAs can be fabricated by several methods,^[^ ^57^ ^]^ including both SPD^[^ ^58^, ^59^ ^]^ and mechanical alloying^[^ ^60^ ^]^ for macro‐scale samples, and magnetron‐sputtering^[^ ^61^, ^62^ ^]^ for nano‐scale prototypic samples: the latter aiming at fast irradiation screening. It is important to emphasize that not all nanocrystalline HEAs and RHEAs can be considered radiation‐tolerant for fusion applications^[^ ^63^, ^64^, ^65^ ^]^ and detailed studies should be carried out for each specific alloy under consideration. The topic RHEAs for nuclear fusion applications has attracted significant attention by the metallurgy and materials science communities in the past year.^[^ ^66^, ^67^, ^68^, ^69^, ^70^, ^71^, ^72^, ^73^, ^74^, ^75^, ^76^ ^]^

Recently, two particular RHEAs systems have attracted the attention of the fusion materials community: the W–Ta–Cr–V^[^ ^65^, ^77^ ^]^ and the W–Ta–Cr–V–Hf.^[^ ^65^, ^78^ ^]^ In the first quaternary system, the W_38_Ta_36_Cr_15_V_11_ RHEA (in at.%), demonstrated superior radiation resistance compared to nanocrystalline W,^[^ ^41^, ^42^, ^43^, ^44^ ^]^ particularly in mitigating displacement damage formation such as dislocation loops, yielding negligible irradiation hardening assessed via nanomechanical testing. On the downside, this W_38_Ta_36_Cr_15_V_11_ RHEA experienced radiation‐induced precipitation (RIP), characterized by the formation of Cr‐V rich precipitates at a radiation dose of 8 dpa (displacement‐per‐atoms). In the second system, irradiation testing on the nanocrystalline RHEA W_29_Ta_42_Cr_5_V_16_Hf_8_ (in at.%) revealed that neither dislocation loops nor precipitates have formed after a dose of 10 dpa.^[^ ^78^ ^]^ Although such recent studies on these two RHEAs show that increased chemical complexity enhances radiation resistance and stability in harsh environments,^[^ ^65^, ^77^, ^78^ ^]^ increasing the number of alloying elements in any RHEA system complicates their bulk fabrication, crucial for practical engineering applications: for example, the low‐melting point of Cr impairs the feasibility for the fabrication of both W_38_Ta_36_Cr_15_V_11_ and W_29_Ta_42_Cr_5_V_16_Hf_8_ RHEAs. Therefore, a key question arises: can the number of alloying elements be reduced in these RHEA systems without compromising their high radiation resistance?

In this study, we show that it is feasible to simplify the quinary RHEA system, the W–Ta–Cr–V–Hf, to a ternary refractory system, the W–Ta–V, resulting in the development of a novel nanocrystalline HCRA, the W_53_Ta_42_V_5_ (in at.%). A recent computational study indicated the WTaV system as a potential and most‐promising low‐activation HCRA with superior radiation resistance when compared with recent RHEAs proposed for application in irradiation environments.^[^ ^79^ ^]^ We demonstrate that the new ternary HCRA not only retains the irradiation resistance observed previously for more complex RHEAs,^[^ ^65^, ^77^, ^78^ ^]^ but also surpasses the irradiation resistance of the binary ultrafine‐grain (UFG) W_44_Ta_56_ (in at.%) highly‐concentrated binary alloy previously proposed as an alternative to W in fusion applications.^[^ ^80^ ^]^ Through detailed post‐irradiation analysis, Atomistic Monte‐Carlo (AMC) simulations, and machine‐learning‐driven molecular dynamics simulations, we investigate the underlying mechanisms of irradiation resistance in this new ternary system, focusing on the role of minor V additions to the irradiation resistance output. We show both theoretically and experimentally that small additions of V drastically changes the non‐irradiation resistant binary WTa system by forming a new irradiation resistant alloy – the nanocrystalline ternary WTaV HCRA. In addition, we demonstrate for the first time that the element Cr is not needed for RHEAs in the context of fusion applications, opening an unprecedented pathway for the synthesis of these novel metallic alloys in bulk and large‐scale quantities. Despite the growing body of literature on RHEAs,^[^ ^53^, ^54^, ^55^, ^56^, ^66^, ^67^, ^68^, ^69^, ^70^, ^71^, ^72^, ^73^, ^74^, ^75^, ^76^ ^]^ the development of W‐Ta‐V‐based compositions remains unexplored. This study addresses this critical gap by introducing a novel W‐Ta‐V HCRA, leveraging the unique properties of W, Ta, and V to achieve an optimal balance of high melting point and irradiation resistance. Furthermore, the reduction in alloying elements offers significant advantages in simplifying manufacturing processes without compromising material performance. By tailoring these properties for extreme environments, this work provides a unique contribution to the field of new materials for nuclear fusion applications.

## 2 Results and Discussion

### 2.1 Morphological Stability Under Extreme Conditions

Heavy ion irradiation with in situ TEM allowed for the comparison of two different HCRAs within the ternary system W–Ta–V, the W_53_Ta_42_V_5_ and the W_53_Ta_44_V_3_. It is important emphasizing that the major objective of the study was to identify the role of the element V in these two alloys' response to both high‐temperature annealing (maximum temperature was 1173 K) and high‐dose irradiation at high‐temperatures (maximum average dose was 20 dpa at an irradiation temperature of 1073 K).

**Figure 1:**
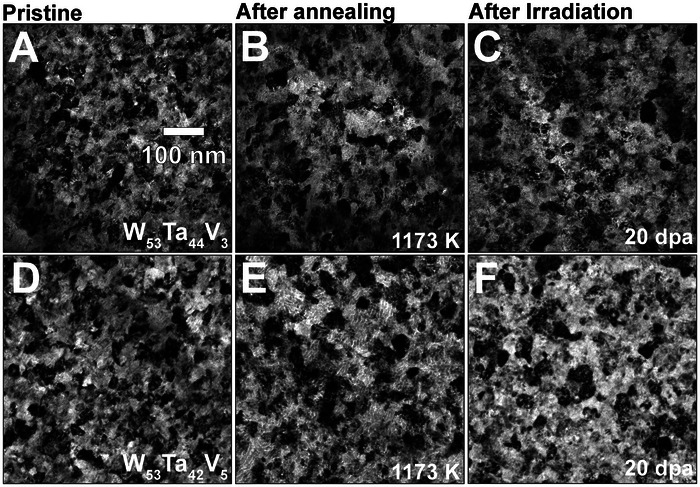
Response to annealing and irradiation | BFTEM micrographs taken at pristine condition, after annealing (at 1173 K) and after irradiation (at 1073 K) are shown in **(**A–C)and (D–F), respectively for the W_53_Ta_44_V_3_ and the W_53_Ta_42_V_5_ HCRAs.

Bright‐Field TEM (BFTEM) micrographs of the W_53_Ta_44_V_3_ and the W_53_Ta_42_V_5_ recorded at three different conditions – pristine, after annealing at 1173 K, and after irradiation at 1073 K – are shown in **Figure** **1**. Before both irradiation and annealing (Figure 1A,D), these alloys exhibited equiaxed grains within the nanocrystalline regime (i.e., <100 nm). Apart from the chemical composition, no major differences between these two HCRAs were identified. By analyzing the BFTEM micrographs after annealing (Figure 1B,E) and irradiation (Figure 1C and 1F), it was noticeable that the W_53_Ta_44_V_3_ HCRA experienced a modest grain growth, particularly noted after 20 dpa of irradiation. Conversely, the W_53_Ta_42_V_5_ HCRA was seemingly unaltered after both annealing and irradiation. A quantitative analysis of average grain sizes as a function of the conditions studied in this work is shown in **Table** **1** where the error bar represents the standard error of the mean.

**Table 1:** Average grain size of HCRAs under pristine, annealed, and irradiated conditions.

Alloy Condition	53W‐42Ta‐3V [at.%]	53W‐44Ta‐5V [at.%]
Pristine (0 dpa)	19.2 ± 0.9	25.9 ± 1.0
Annealed at 1173 K (0 dpa)	27.5 ± 0.9	29.1 ± 1.4
Irradiated at 1073 K (20 dpa)	29.4 ± 1.3	27.3 ± 2.1

These results exhibited in Figure 1 are better comprehended considering the challenges on the application of nanocrystalline alloys in extreme environments. These challenges are intrinsically related with the (thermodynamic and morphological) stability of nanocrystalline materials^[^ ^81^, ^82^, ^83^ ^]^ when subjected to exposure in both irradiation^[^ ^84^ ^]^ and high‐temperatures.^[^ ^85^ ^]^ In this context, the primary adverse consequence of such exposure is grain growth as numerous material properties stem from the average grain size. To date, and according to Zhang et al.,^[^ ^84^ ^]^ a limited number of studies addressed the grain size stability of nanocrystalline metallic alloys, and in this way, existing studies on this field are primarily focused on the behavior of nanocrystalline elemental metals. Herein, it is consolidated that elemental metals in their nanocrystalline form experience significant grain growth under heating/annealing and accelerated grain growth under irradiation over a wide range of temperatures. With respect to nanocrystalline elemental metals exposed to extreme conditions, it is important to emphasize that theoretical models were already developed and experimentally validated to explain such observations.^[^ ^86^, ^87^, ^88^, ^89^ ^]^

Nanocrystallinity allows achieving higher radiation tolerance within the scope of novel nuclear materials.^[^ ^42^, ^90^, ^91^, ^92^ ^]^ This is due to the presence of a greater number of interfaces (namely grain boundaries) that enhances the capacity for radiation‐induced crystalline defects to be absorbed and recombine effectively at these site‐specific dependencies.^[^ ^90^, ^91^ ^]^ This is historically known and defined as “sink efficiency”, and for a metal, the sink efficiency increases exponentially with decreasing average grain size.^[^ ^90^, ^91^ ^]^ When nanocrystalline alloys undergo grain growth or recrystallization due to energetic particle irradiation, their radiation tolerance is compromised. In such cases, the challenges that affect W for fusion reactors^[^ ^15^, ^16^, ^17^, ^41^, ^42^, ^43^, ^44^ ^]^ also become relevant for both RHEAs and novel HCRAs. Concerning the current state of research on RHEAs, which are undergoing intensive development for fusion applications, it is worth noting that although there are limited studies on grain size stability under irradiation and high‐temperature conditions, recent research reveals two critical findings: i) not all nanocrystalline HEAs exhibit irradiation tolerance,^[^ ^63^, ^64^ ^]^ and ii) certain RHEAs demonstrate superior performance under irradiation at high‐temperatures than both coarse‐grained and nanocrystalline W.^[^ ^41^, ^42^, ^43^, ^44^, ^77^, ^78^, ^93^ ^]^

Precipitation was observed in the quaternary nanocrystalline RHEA – W_38_Ta_36_Cr_15_V_11_^[^ ^77^ ^]^ – when tested under heavy‐ion irradiation, and although no significant alterations in the grain morphology were observed, such phase evolution effects are signs of degradation of the initially designed alloy. A new quinary RHEA^[^ ^78^ ^]^ – W_29_Ta_42_Cr_5_V_16_Hf_8_^[^ ^78^ ^]^ – was recently designed and synthesized. In terms of grain morphology after irradiation, the addition of Hf was associated with grain refinement/fragmentation during both annealing and irradiation, therefore indicating some grain instabilities that deserve further investigations. Although these previous works indicated that higher phase stability was achieved by increasing the chemical complexity, morphological changes were still observed in the microstructures of both alloys after irradiation. In comparison with both the W_38_Ta_36_Cr_15_V_11_^[^ ^77^ ^]^ and the W_29_Ta_42_Cr_5_V_16_Hf_8_^[^ ^78^ ^]^ RHEAs, the results presented in Figure 1 show that new HCRAs with lower chemical complexity – W_53_Ta_42_V_5_ and the W_53_Ta_44_V_3_ – can be synthesized in its nanocrystalline form and still preserve high microstructural stability after both high‐temperature annealing and irradiation exposure observed in the RHEAs with more alloying elements. The average grain sizes measured at pristine, after annealing at 1173 K and after irradiation at 1073 K and up to 20 dpa attests these findings. These results presented in Table 1 suggest that high‐temperature annealing drives grain growth due to the removal of voided grain boundaries manifested as nanoporosity in the pristine samples after magnetron‐sputtering deposition:^[^ ^98^ ^]^ evidence for such nanoporosity is presented in the Supporting Information. Conversely, irradiation at 1073 K after annealing indicates no detectable grain growth up to the tested dose of 20 dpa. Therefore, we can conclude that both W–Ta–V HCRAs exhibit a high degree of irradiation tolerance in terms of grain stability, as evidenced by the absence of growth and/or recrystallization after high‐dose irradiation.

**Figure 2:**
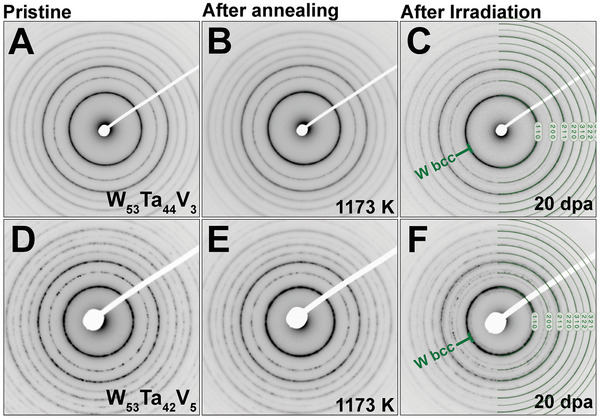
Phase stability to annealing and irradiation | SAED patterns collected at pristine condition, after annealing (at 1173 K) and after irradiation (at 1073 K) are shown in (A–C) and **(**D–F), respectively for W_53_Ta_44_V_3_ and the the W_53_Ta_42_V_5_ HCRAs. Additional rings were noted following irradiation, with a higher intensity observed in the W_53_Ta_42_V_5_ HCRA (in **F**) compared to the W_53_Ta_44_V_3_ HCRA (in **C**). Indexing was performed with data available in literature.^[^ ^94^, ^95^, ^96^, ^97^ ^]^

The phase stability of both W–Ta–V HCRAs was also investigated with the Selected Area Electron Diffraction (SAED) technique during the in situ TEM experiments of high‐temperature annealing and heavy‐ion irradiation. **Figure** **2**A,D show the SAED patterns collected on the two different W–Ta–V HCRAs in their pristine condition; both alloys were indexed to have BCC crystalline structure matching W standards.^[^ ^94^, ^96^ ^]^ The phase of both alloys remained unaltered after high‐temperature annealing, as noted in Figure 2B,E. After irradiation up to 20 dpa, some additional Debye‐Scherrer rings appeared in the SAED patterns of both alloys, as shown in Figure 2C,F, although in the alloy with higher V content (Figure 2C), these rings are of lower intensity. These extra‐rings could not be identified to any specific crystal structure or symmetry. It is important to emphasize that recently, these extra rings were also observed in the quinary W_29_Ta_42_Cr_5_V_16_Hf_8_ RHEA after irradiation and as such, they were not indexed to any known phase.^[^ ^78^ ^]^

The microstructure of both W–Ta–V alloys, as depicted in Figure 1, reveals no phase transformation following high‐temperature annealing or irradiation. The appearance of additional rings in the SAED patterns, as shown in Figure 2, after a dose of 20 dpa, suggests the potential occurrence of phase instabilities through precipitation, thus underscoring the need for further nanoscale post‐irradiation screening with analytical microscopy methods.

### 2.2 Chemical Stability Under Extreme Conditions

**Figure 3:**
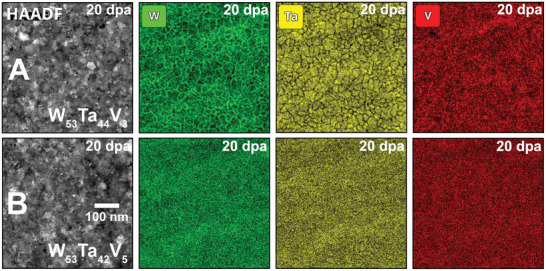
Nanoscale chemistry of the WTaV HCRAs after irradiation at 20 dpa | High‐magnification STEM‐EDX mapping collected from both W_53_Ta_42_V_5_ (top row) and the W_53_Ta_44_V_3_ (bottom row) HCRAs reveal W segregation and Ta depletion at the nanocrystalline grain boundaries upon decreasing the V content in the W–Ta–V system. The W_53_Ta_42_V_5_ HCRA neither exhibit radiation‐induced segregation nor radiation‐induced precipitation or phase instabilities/transformations, indicating a high‐radiation tolerance at 20 dpa.

Scanning Transmission Electron Microscopy coupled with Energy Dispersive X‐ray (STEM‐EDX) spectroscopy mapping was carried out to assess the local chemistry of both W–Ta–V HCRAs after heavy‐ion irradiation at 1173 K up to 20 dpa. **Figure** **3**A,B show the microstructure of the irradiated W_53_Ta_42_V_5_ and the W_53_Ta_44_V_3_ HCRAs, respectively, using both High‐Angle Annular Dark‐Field (HAADF) and EDX elemental maps. While the W_53_Ta_44_V_3_ HCRA clearly indicates the occurrence of W segregation and Ta depletion at grain boundaries, no such segregation was observed or detected in the W_53_Ta_42_V_5_ HCRA. Complimentary STEM‐EDX maps for both pristine and annealed‐only conditions are shown in the Supporting Information.

The nanoscale analytical assessment presented in Figure 3 for both alloys after irradiation suggests that lower concentrations of V reduce the chemical stability of the W–Ta–V system. Interestingly, the W_53_Ta_42_V_5_ HCRA not only exhibit high morphological stability under irradiation (Figure 2), preventing grain growth or recrystallization up to a dose of 20 dpa at 1073 K, but also presents high chemical stability given the absence of grain boundary segregation under the irradiation conditions studied. These results shed light on the behavior that these HCRAs – manifested by the reduction on the number of alloying elements when compared with conventional RHEAs^[^ ^77^, ^78^ ^]^ – can have their radiation response tailored as a function of the concentration of V. Therefore, further investigation is needed to explore the specific role of V in the context of the W–Ta–V system. While Atom Probe Tomography (APT) could provide detailed insights into atomic‐scale ordering or precipitation following irradiation, the preparation of specimens from these 70 nm thin films proved challenging.

### 2.3 The Role of V in the Stability and Radiation Response of Chemically Simplified HCRAs

**Figure 4:**
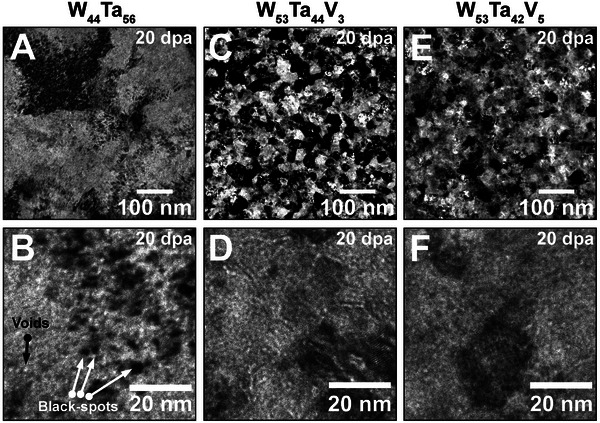
Comparison between binary WTa alloy and ternary WTaV HCRAs at 20 dpa | Defects after irradiation are observed in the UFG W_44_Ta_56_Ta alloy as shown in the BFTEM micrograph in (A) and the underfocused BFTEM micrograph in (B): this binary alloy exhibit both voids and black‐spots as particularly shown in (B). Grain morphology for both irradiated W_53_Ta_44_V_3_ and W_53_Ta_42_V_5_ HCRAs are shown in the BFTEM micrographs in (C) and (E): both alloys are stable in terms of grain‐size after 20 dpa. In addition, no irradiation‐induced black‐spots were noticeable after irradiation. Underfocused BFTEM was used to better resolve voids in the ternary HCRAs as shown in micrographs (D) and (F): voids were resolvable in the W_53_Ta_44_V_3_ HCRA, but in the W_53_Ta_42_V_5_ HCRA they are smaller and less uniformly distributed, thus in the limit of detection by the TEM.

To investigate the role of V on the radiation response of the W–Ta–V system, additional high‐temperature irradiation experiments were conducted with a binary UFG W_44_Ta_56_ alloy that was synthesized under similar deposition conditions to the HCRAs, but containing no V. The microstructure of the UFG W_44_Ta_56_ alloy after 20 dpa is shown in both low‐ and high‐magnification BFTEM micrographs in **Figure** **4**A,B, respectively. After irradiation, this binary alloy exhibited extensive nucleation defects in a form of voids and black‐spots (displacement damage), as evident in the BFTEM micrograph presented in Figure 4B. Yi et al. reported an extensive chain of irradiation‐induced defects to nucleate and evolve in a coarse‐grained binary alloy W‐5Ta (in wt.%) – a terminal solid solution – at doses as low as 1.2 dpa.^[^ ^80^ ^]^ The findings of Yi et al.,^[^ ^80^ ^]^ together with our results for the UFG W_44_Ta_56_, indicate that W‐Ta binary alloys experience radiation damage at low doses, regardless of grain size (coarse or ultrafine) or composition (highly concentrated or terminal solid solution).

Similarly to the irradiated binary UFG W_44_Ta_56_ alloy, the microstructures of both the W_53_Ta_42_V_5_ and the W_53_Ta_44_V_3_ HCRAs after the same 20 dpa heavy‐ion irradiation are shown in Figure 4C‐D and 4E–F, respectively, and utilising BFTEM imaging. The binary alloy as‐synthesized has larger grain sizes, pertaining to the UFG regime, when compared with the ternary HCRAs, which are nanocrystalline. While some voids were observed in intra and transgranular regions of the W_53_Ta_44_V_3_ HCRA (Figure 4D), voids were resolvable in the W_53_Ta_42_V_5_ HCRA only in the transgranular sites with lower areal densities, as shown in Figure 4F. **Table** **2** shows the quantification of voids for all three alloys after irradiation at 20 dpa. In Table 2, the error bar represents the standard error of the mean. The results indicate that nanocrystalline HCRAs exhibit superior performance compared to UFG binary alloy in terms of their resistance to the formation of black‐spots and voids under irradiation, directly suggesting that the small additions of the element V, increases significantly the radiation response of the binary alloy. In terms of void nucleation and growth, the alloy W_53_Ta_42_V_5_ HCRA outperforms both its ternary counterpart with less V and the binary UFG W_44_Ta_56_ alloy. However, it is important noting that in the specific conditions of our study, nanocrystallinity was only achieved in the W–Ta system when V was added as an alloying element: hypothetically, this indicates that a nanocrystalline binary WTa alloy without V could exhibit less build‐up of voids and black‐spots compared with our study with the UFG W_44_Ta_56_ alloy, see Figure 4A,B. The dual role of V as a grain refiner and enhancer of radiation resistance in the W–Ta system highlights its potential in designing advanced HCRAs for fusion applications, warranting further investigation following this study.

**Table 2:** Average void size (diameter) and areal densities for all samples after irradiation up to a dose of 20 dpa at 1073 K.

Irradiated Alloy	Void Size [nm]	Areal Density [#·nm^−2^]
W_44_Ta_56_	2.37 ± 0.01	1.8 ± 0.2 × 10^−2^
W_53_Ta_44_V_3_	1.65 ± 0.01	1.3 ± 0.1 × 10^−1^
W_53_Ta_42_V_5_	1.19 ± 0.01	8.3 ± 0.8 × 10^−2^

Based on our observations among all studied alloys irradiated up to 20 dpa at 1073 K, the HCRAs exhibit both smaller average void sizes and average areal density than the binary UFG W_44_Ta_56_ alloy: already an indicative of higher radiation tolerance for the ternary alloys with V when compared with the binary alloy. Upon increasing the V content from 3 to 5 at.%, smaller voids were observed in the W_53_Ta_42_V_5_ HCRA and with an areal density distribution one order of magnitude lower than the W_53_Ta_44_V_3_ HCRA. The radiation tolerance of these new HCRAs is better evaluated when recent data on in situ TEM ion irradiation of relevant fusion materials is taken into consideration. Under similar irradiation temperatures and lower doses to this present work, in situ TEM with He implantation revealed He bubbles with an average diameter of 6.6 nm and an areal density of 2.7 × 10^−2^ bubbles·nm^−2^ for bulk W.^[^ ^99^, ^100^ ^]^ For UFG W, the average diameter of bubbles was reported to be 6.4 nm with an areal density of 7.5 × 10^−2^ bubbles·nm^−2^.^[^ ^101^ ^]^ The previous W_38_Ta_36_Cr_15_V_11_ RHEA exhibited He bubbles of around 2.2 nm in diameter with an areal density of 1.5 × 10^−1^ bubbles·nm^−2^.^[^ ^77^ ^]^ Both average void size and areal density for the HCRAs investigated are similar to bulk W,^[^ ^99^, ^100^ ^]^ UFG W^[^ ^101^ ^]^ and previous chemically‐complex quaternary RHEAs.^[^ ^77^ ^]^ The voids sizes are smaller than more complex HCRAs and pure W.^[^ ^77^ ^]^ The results in this work show that the existing W–Ta system can have its radiation tolerance significantly enhanced by adding the element V in small concentrations: from 3 to 5 at.% of of V into WTa, a reduction in both areal density and size of voids is recorded. No radiation‐induced segregation (i.e., better phase stability) – is noted for the HCRA with higher V content.

### 2.4 Chemical Short‐Range Order Within the W–Ta–V System

To gain a deeper understanding of the important role of V on radiation‐induced stability in the considered HCRAs, an AMC modelling approach based on Density Functional Theory (DFT) and Cluster Expansion Hamiltonian (CEH) methods, developed recently for multi‐component system,^[^ ^102^, ^103^, ^104^, ^105^ ^]^ has been employed to predict the CSRO of the three different pairs (W‐Ta, W‐V, and Ta‐V) and thermodynamic properties of both W_53_Ta_44_V_3_ and W_53_Ta_42_V_5_ alloys.

**Figure 5:**
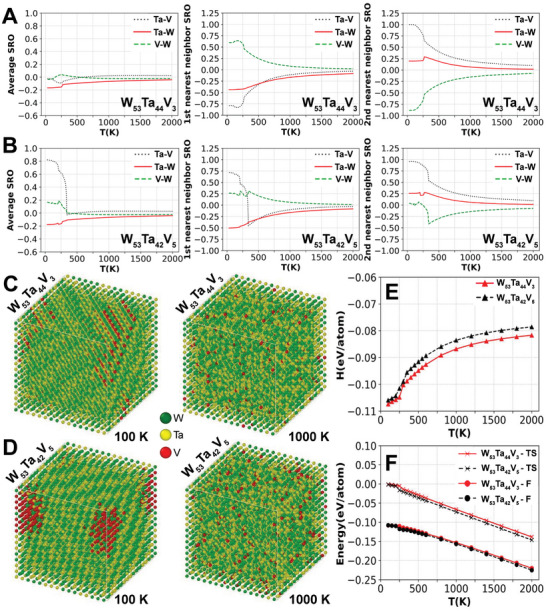
DFT‐CEH based AMC simulations for W_53_Ta_44_V_3_ and W_53_Ta_42_V_5_ | The predicted Warren‐Cowley chemical short‐range order parameters (average, 1NN and 2NN) for different pairs of atoms (W‐Ta: blue; W‐V: red and Ta‐V; yellow) as a function of temperature for W_53_Ta_44_V_3_ in (A) and W_53_Ta_42_V_5_ in (B). Atomic structures obtained from AMC simulations at 100K and 1000K are shown for W_53_Ta_42_V_3_ in (C) and W_53_Ta_44_V_5_ in (D). The enthalpy of mixing (E), configuration entropy and free energy (F) calculated as a function of temperature for the two considered HCRA alloys.

In agreement with our previous investigations in quinary alloys containing W, Ta and V,^[^ ^104^, ^105^ ^]^ the calculated Warren‐Cowley CSRO parameter of the W‐Ta pair averaged over first nearest‐neighbor (1NN) and second nearest‐neighbor (2NN) in the BCC system is negative as a function of temperature in both W_53_Ta_44_V_3_ and W_53_Ta_42_V_5_ as shown in **Figures** **5**A and 5B, respectively. This common behavior resulting from the dominance of chemical bonding between W and Ta atoms in the 1NN and the repulsion at the 2NN can be explained by the negative enthalpy of mixing, which has been predicted in all ranges of composition in the binary W–Ta system.^[^ ^106^ ^]^ In particular, at the composition closer to equiatomic, the above finding is related to the existence of a B_2_ phase in a simple interpretation of the binary W–Ta phase diagram^[^ ^107^ ^]^ whereas more accurate DFT calculations predicted the stability of an orthorhombic A_6_B_6_ phase.^[^ ^106^ ^]^

The average CSRO of W‐V pair in the two ternary alloys with rich W and Ta concentration is, however, quite different to those of W‐Ta as depicted in Figure 5A,B although a similar trend of negative enthalpy of mixing has also predicted in the binary W‐V system.^[^ ^106^ ^]^ Due to low V concentration, the CSRO W‐V pair is found to be strongly positive for the 1NN, while it is mostly negative for the 2NN as a function of temperature. There is, however, a significant difference between the two alloys with different V concentrations. Namely, at low temperatures (T < 250K), the 2NN CSRO between W‐V changes from negative to positive for the W_53_Ta_42_V_5_ HCRA, whereas it continues to be negative for the W_53_Ta_44_V_3_ HCRA. This means that for the latter case, V atoms are strongly to be present in the 2NN shell of a W atom, while in the former case they tend to remain far from W.

In a consistency with the above analysis for the 2NN CSRO of W‐V, the second interesting difference between the two alloys considered W_53_Ta_42_V_5_ and W_53_Ta_44_V_3_ is found in the 1NN CSRO of Ta‐V pairs. As both Ta and V are transition metals in group 5 of the periodic table, the enthalpy of mixing is expected to be positive in the binary Ta‐V system and, accordingly, the 1NN CSRO should be positive indicating the segregation tendency between Ta and V. In a strong variance with the binary alloy system, the CSRO of the 1NN Ta‐V pair in the W_53_Ta_44_V_3_ HCRA is negative for all temperatures demonstrating the important effect of W concentration on the chemical SRO between Ta and V. In the W_53_Ta_42_V_5_ HCRA, a similar negative trend for the 1NN CSRO of Ta‐V pair is observed for temperatures higher than about 350 K, but it becomes positive for T < 350K. This dramatic change yields different behavior of the average CSRO parameter for Ta‐V of W_53_Ta_42_V_5_ in comparison with those of W_53_Ta_44_V_3_ at low temperature. It is worth mentioning that our comparison of the predicted CSRO at low‐temperature for the 1NN Ta‐V pairs and those of the 2NN for W‐V pairs in the two considered HCRAs serves as a crucial precursor to differentiate their properties systematically as a function of temperatures including at higher temperatures. For instance, at T = 1200 K, our calculated CSRO for 1NN Ta‐V and 2NN W‐V are found to be −0.0803 ± 0.0005 and −0.1283 ± 0.0005, respectively, in W_53_Ta_44_V_3_ that are stronger negative than the corresponding values of −0.0736 ± 0.0006 and −0.1194 ± 0.0003, respectively, found in the HCRA with higher V, i.e., the W_53_Ta_42_V_5_. Stronger negative CSRO at higher temperatures (and irradiation as it enhances solid‐state diffusion) suggest a tendency for the HCRA to exhibit segregation as observed in the W_53_Ta_44_V_3_ HCRA (see Figure 3B). This segregation is suppressed upon minor additions of V to compose the W_53_Ta_42_V_5_ HCRA, which has a decreased CSRO effect, resulting in increasing the chemical and thermodynamic stability of the random solid solution.

The above analysis of CSRO parameters demonstrates a sensitivity of microstructural stability with respect to the CSRO as a function of both V concentration and temperature in the W–Ta–V system. Figure 5C,D show the results of AMC simulations in a cell with 16.000 atoms obtained at 100 and 1000 K for W_53_Ta_44_V_3_ and W_53_Ta_42_V_5_ HCRAs, respectively. A stronger tendency of ordering surrounding V atoms for the former system was in good agreement with the SAED indexing in W_53_Ta_44_V_3_ after irradiation (see Figure 2F). It is worth emphasising that at high temperatures, the 1NN contribution to the CSRO behavior for V‐Ta pair is dominantly negative and much stronger for W_53_Ta_44_V_3_ than those of W_53_Ta_42_V_5_ as it can be seen from Figure 5,B. Our AMC simulations also indicate the more homogeneous atomic distribution in W_53_Ta_42_V_5_, where voids form at one order of magnitude lower areal density at 20 dpa at high temperature as shown in Table. 2. Finally, our calculations of enthalpy of mixing (Figure 5E), configurational entropy and free energy depicted in Figure 5F showed a more negative enthalpy of mixing for W_53_Ta_44_V_3_ over W_53_Ta_42_V_5_ over all the temperature range mainly due to V‐driven chemical ordering effect in the former HCRA.

It is important to establish the correlation between V‐Ta CSRO with the observed microstructural changes after irradiation for the two W–Ta–V HCRAs with respect to a small variation of V concentration from our AMC simulations. The lower‐V alloy W_53_Ta_44_V_3_ has the radiation‐induced second V‐bcc phase due to anomalous negative average CSRO parameter between V‐Ta while the higher‐V alloy W_53_Ta_42_V_5_ has a dominant single W‐bcc phase with a segregation tendency between V and Ta associated with the positive average CSRO. It is worth emphasizing the important role of V on distinct and low radiation‐induced defect formation properties in comparison with those of W and Ta,^[^ ^108^, ^109^ ^]^ and therefore, providing insight into enhanced radiation resistance in the bcc MEAs W–Ta–V system. Crucially, the prediction of strong change for Ta‐V CSRO properties between W_53_Ta_44_V_3_ and W_53_Ta_42_V_5_ provides an excellent support to the experimental observation of a suppression of voids from the binary W_44_Ta_56_ to the HCRAs as it is shown in Table 2. According to molecular dynamic (MD) simulations, a slow self‐interstitial atom (SIA) and fast vacancy diffusion due to weak binding energies of V dumbbells in comparison with significantly larger ones of W and Ta lead to the closer mobilities of vacancies and SIAs, which enhances defect recombination in the annealing process in W–Ta–V alloys.^[^ ^110^ ^]^ To support these findings, additional AMC simulations have been performed for W_44_Ta_56_ with 0.5% vacancy using the DFT‐CEH, as reported elsewhere.^[^ ^102^ ^]^ A strong presence of voids for this binary system is observed and in good agreement with the experimental observation shown in Figure 4D for W_44_Ta_56_ (see Figure S1, Supporting Information). Importantly, the average void size obtained from AMC simulations equal to 1.5 ± 0.4 nm agrees with the value from the experiment reported in Table 2. A further systematic investigation will be focusing on the defect properties as a function of V alloying concentration in radiation‐induced microstructure stability of W–Ta–V system. Finally, it is interesting to predict new candidate simplified HCRAs with superior radiation resistance from a CSRO behavior similar to those of W–Ta–V HCRAs investigated in this work. One of the candidates would be W–Ti–V alloys for which our AMC simulations were performed based on CEH developed in ref. [104]. It is found that for the W_53_Ti_42_V_5_ alloy the temperature dependence of CSRO between Ti‐V as well as the enthalpy of mixing are similar to those of Ta‐V at the temperature around 250K for W_53_Ta_42_V_5_ leading to a potentially single bcc‐based W phase at high‐temperature region (See Figure S2, Supporting Information). CSRO has been recently linked with potential higher radiation resistance in a HfNbTaTiZr RHEA in a computational study carried out by Mo et al.^[^ ^71^ ^]^ In our present study, the analysis of CSRO in the W–Ta–V ternary system is compared with real experimental results for the first time in HCRAs as candidate fusion materials.

### 2.5 Simulated Radiation Damage Accumulation and Annealing

Radiation damage accumulation on short time scales can be directly estimated with MD simulations.^[^ ^111^ ^]^ This provides detailed information about defect production, clustering, and recombination with atomistic resolution. We simulate the radiation damage buildup in W_44_Ta_56_, W_53_Ta_44_V_3_, and W_53_Ta_42_V_5_ alloys at 1073 K using a newly developed machine‐learned interatomic potential in MD simulations of overlapping collision cascades. With detailed analysis of the defect structure, we gain further insight into the role of V in the radiation tolerance.

Recent MD simulations showed that presence or absence of V in W‐based refractory alloys strongly define their radiation response due to its small size and low mass.^[^ ^79^ ^]^ Two main consequences of adding V were identified:^[^ ^79^ ^]^ 1) the presence of V balances the migration rate of vacancies and self‐interstitial atoms (mainly by lowering the mobility of the latter), leading to enhanced recombination, and 2) the presence of V alters the binding energies of vacancy clusters, causing a shift from preferential formation of voids (without V) to vacancy‐type dislocation loops (in alloys with V). However, these conclusions are based on MD simulations of equimolar compositions of WTaV and other W‐based alloys. It cannot be assumed that the small concentrations of V considered here (3 and 5 at.%) have the same effect as in equimolar WTaV.

**Figure 6:**
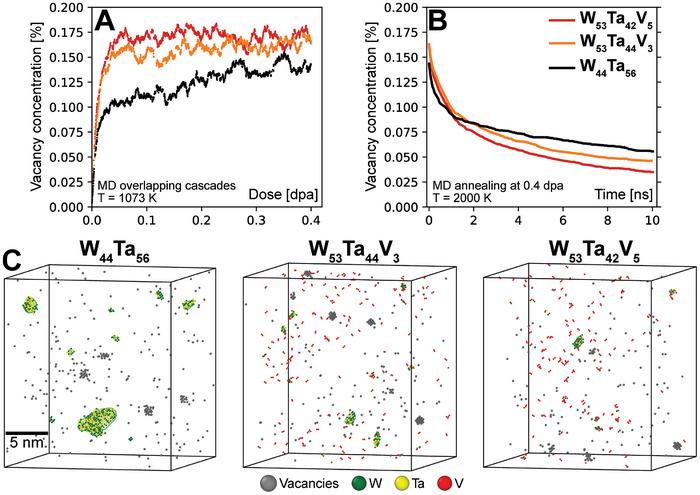
MD simulations of damage accumulation and defect recombination during annealing | Irradiation‐induced vacancy concentrations as functions of dose in the three HCRAs in (A). Vacancy concentrations as functions of time during MD annealing at 2000 K in (B), showing the recombination efficiency of the HCRAs. Snapshots of the defect structure after annealing, showing vacancies (grey atoms), self‐interstitial atoms (colored by element) and dislocation loops (green lines) in (C).

**Figure** **6**A shows the defect concentrations in all three alloys as functions of dose at 1073 K. Even though only relatively low doses (<1 dpa) are computationally accessible, Figure 6A reveals a clear difference in the damage buildup between W_44_Ta_56_ and the 3 and 5 at.% V‐containing ternary alloys. In the binary W_44_Ta_56_, the defect concentrations remain lower, but continues to increase as a function of dose. In the V‐containing alloys, the defect concentrations rapidly increases and then saturates, showing no further change in the overall defect concentration as the dose increases. Qualitative insight into long‐term microstructure evolution is gained by annealing all three alloys in MD at 2000 K for 10 ns at the dose of 0.4 dpa. The defect concentrations as function of time are shown in Figure 6B. With increasing V concentration, the recombination rate of defects drastically increases. Figure 6B reveals the key finding from the MD simulation that directly support the experimental observations of reduced void growth with increasing V concentration. The increased recombination efficiency with the addition of V is in line with the previous MD results, although it is remarkable that a V concentration as low as 5 at.% causes an effect comparable to that of equimolar WTaV.^[^ ^79^ ^]^

Figure 6C shows snapshots of the defect microstructure of the three alloys at 0.4 dpa after MD annealing at 2000 K. Only vacancies, self‐interstitials atoms, and dislocation lines are shown. The clear difference in defect clusters, in particular interstitial‐type clusters, highlight the difference between the radiation response between the binary W–Ta and ternary W–Ta–V HCRAs. In W–Ta, the self‐interstitials produced during irradiation are more mobile than vacancies and cluster to form interstitial‐type dislocation loops with the energetically preferred Burgers vector 1/2〈111〉. This large dislocation loop acts as a sink for defects in the MD simulation, leaving excess vacancies behind to cluster and eventually form voids. It is worth noting the MD simulations consider a single crystal. In the experimental samples the dislocation loops likely move and annihilate at grain boundaries, with the same consequence of leaving less mobile vacancies behind to form voids. In the W_53_Ta_44_V_3_ and W_53_Ta_42_V_5_ alloys, the reduced mobility of interstitials prevents formation of large interstitial clusters. Instead, vacancies and interstitials move at a similar rate, leading to formation of only small clusters and efficient recombination. These observations are directly in line with too‐small size of voids and low areal densities in W_53_Ta_42_V_5_ in TEM. Figure 6C shows that there is some vacancy clustering and formation of nano‐sized voids in all alloys, the void‐like clusters are slightly larger and more frequent in W_44_Ta_56_ and W_53_Ta_44_V_3_ than in W_53_Ta_42_V_5_. Although not yet computationally permitted, higher doses, and longer time scales closer to the experiments would likely emphasize these differences due to the mechanisms outlined above and bring the results even closer to the TEM‐measured void distributions. Furthermore, it is imperative to acknowledge that the dose rate exhibited by MD simulations may be substantially higher than that of actual irradiation experiments, potentially by an order of magnitude or more. Consequently, the MD simulation results presented in this study are intended to serve as a means of interpreting the mechanisms by which WTaV HCRAs demonstrate superior radiation tolerance in comparison to binary WTa.

## 3 Conclusion

We have demonstrated by means of irradiation‐testing and characterization experiments that new HCRAs can be developed and their radiation response can be tailored as a function of minor modifications in the alloys' chemistry. Two new nanocrystalline HCRAs were synthesized and investigated in this work: W_53_Ta_42_V_5_ and W_53_Ta_44_V_3_. Atomistic simulations of short‐range order in these bcc HCRAs unravelled the significant role of V alloying effects on radiation‐induced micro‐structural stability and defect formation properties in W–Ta–V via a systematic investigation of dependence in CSRO for the Ta‐V in 1NN and those for the W‐V in 2NN as a function of V concentration and temperature. Minor variations in V content lead to differences in phase stability, segregation behavior, and radiation response that were experimentally observed and computationally validated. MD simulations with a machine‐learned interatomic potential revealed the mechanisms behind the drastic change in radiation response caused by minor concentrations of V. The transition from RHEAs to chemically simplified HCRAs has the potential to greatly influence the quest for materials with high radiation tolerance for the practical application in future nuclear fusion reactors. In addition, the WTaV HCRAs studied in this work address the concerns of materials' activation whilst under in‐service in a fusion reactor: they both present reduced activation when comparing with existing RHEAs so far investigated. This potential arises from the reduction in chemical complexity, achieved by decreasing the number of alloying elements without jeopardizing the high‐stability inherent to HEAs with four or more constituents. This simplification is aimed at facilitating the metallurgical processes for synthesizing these alloys in bulk and at larger scales, which is a necessary step for achieving both technological readiness and commercialization of materials for future fusion reactors. Therefore, this study lays the groundwork for future investigations into defect properties as a function of V alloying concentration and the exploration of new candidate multi‐element alloys with enhanced radiation resistance and simplified‐chemistry, the latter manifested by a reduction on number of alloying elements, thus deviating from the original high‐entropy alloy concept^[^ ^48^ ^]^ to devise a new and optimized way to think and design the future materials for nuclear fusion.

It is important to acknowledge that materials for nuclear fusion reactors must endure irradiation doses exceeding 20 dpa and temperatures above 1273 K. The experiments conducted in this study provide preliminary evidence that the HCRA alloys investigated could be viable candidates for further evaluation in materials test reactors (MTRs). The prototypic approach of using in situ TEM heavy ion irradiation serves as an effective method for rapidly screening alloy candidates, enabling the identification of promising materials for MTR testing while minimizing costs and avoiding the challenges associated with handling activated specimens.

## 4 Experimental Section

### Synthesis of the WTa and WTaV Alloys

Metallic alloys in the binary system W–Ta and the ternary system W–Ta–V were prepared in a form of thin solid films using the magnetron‐sputtering deposition technique from elemental targets with nominal 99.99 at.% purity. The deposition was performed at room temperature, 3 mTorr of pressure, and with no bias voltage. Multiple thin solid films were deposited on pure NaCl (100) substrates from Hilger Crystals, Inc. After deposition, the substrates were dissolved in a deionized water and pure ethanol solution, and the floating films were placed onto pure Mo mesh grids for TEM analysis and irradiation. The thickness of the deposited alloys was estimated to be ≈ 70 nm using profilometry.

### Heavy‐Ion Irradiations with in situ TEM

All the alloys investigated in this work were irradiated with 1 MeV Kr heavy‐ions with in situ TEM at the Intermediate Voltage Electron Microscope (IVEM) Tandem facility located at the Argonne National Laboratory. Prior irradiation, the samples were subjected to 10 min of annealing at 1173 K using a Gatan double‐tilt heating holder (furnace‐type). Irradiations were then carried out at temperature of 1073 K with a Kr flux measured to be 1.25 × 10^12^ ions cm^−2^ s^−1^. BFTEM micrographs and SAED patterns were recorded before and after irradiation. During irradiation, the samples were under constant electron‐beam monitoring using a Hitachi TEM 9000 operating a LaB_6_ filament at 300 keV. Multiple videos were recorded during both annealing and irradiation and can be found in the Supporting Information. Fluence‐to‐dpa calculations were performed using the Stopping and Range of Ions in Matter (SRIM) Monte Carlo code version 2013.^[^ ^112^ ^]^ For these calculations, a procedure described by Stoller et al. was used,^[^ ^113^ ^]^ which consists in using the Quick‐Damage calculation mode setting the displacement energies as 40 eV. The alloys in this study were subjected to 1 h of irradiation, achieving a fluence of 4.50 × 10^15^ ions cm^−2^, equivalent to an average of ≈20 dpa across the assumed 100 nm sample thicknesses and the SRIM calculated default densities (in g cm^−3^). The irradiation dose profile as a function of thickness, as well as the implantation profile are shown in the Supporting Information. It was important to remind that this calculation methodology was sensitive to the chosen displacement energy values. 40 eV was used to stay consistent with previous publications,^[^ ^77^, ^78^ ^]^ as SRIM's default value of 25 eV for non‐alloyed refractory elements like W, Ta, and V likely underestimates the dose in dpa. Further experimental and computational studies were needed to determine accurate threshold displacement energies for refractory transition metals in high‐entropy alloys or terminal solid solutionconfigurations.

### Post‐Irradiation Screening with Conventional and Analytical S/TEM

Following irradiation, samples were allowed to cool within the TEM's vacuum environment by deactivating the external heating supply provided by GATAN. After cooling, these samples were removed for further post‐irradiation carried out with a FEI Titan 80–300 STEM located at the Los Alamos National Laboratory, a Thermo Fisher Talos F200X located at the Montanuniversität Leoben, and a FEI Titan 80‐300 STEM located at the Pacific Northwest National Laboratory. Post‐irradiation characterization was performed using STEM mode and the High‐Angle Annular Dark‐Field (HAADF) and Bright‐Field (BF) detectors, as well as Energy Dispersive X‐ray (EDX) spectroscopy that was used for elemental mapping of the unirradiated and irradiated alloys. SAED was used to assess the phase stability of the alloys in pristine condition, as well as after annealing at 1173 K and after irradiation at 1073 K. SAED pattern indexing was performed with data available in the ICSD database,^[^ ^96^ ^]^ more specifically: ICSD‐52268 for W (BCC),^[^ ^94^ ^]^ ICSD‐53786 for Hf (HCP),^[^ ^95^ ^]^ and ICSD‐171003 for V (BCC).^[^ ^97^ ^]^ Pertaining the characterization of irradiation‐induced defects, it was important emphasising that the observation of voids in all alloys was dependent on the spatial resolution of the electron‐microscopes that was around 0.25 nm.

### Atomistic Monte Carlo and Cluster Expansion Modelling

In this study, AMC simulations were performed using CEH formalism which had been recently developed for studying the phase stability of compositionally complex and multi‐component systems as a function of temperature and irradiation damage for different structural materials based on W‐based^[^ ^102^, ^104^, ^105^, ^106^, ^114^ ^]^ and Fe‐based^[^ ^115^, ^116^ ^]^ alloys. The CEH can be written in the following formula:^[^ ^102^ ^]^

(1) $${\Delta H}_{mix}^{CEH}\left(\vec{\sigma }\right)=\sum_{\omega ,n,s}J_{\omega ,n}^{\left(s\right)}m_{\omega ,n}^{\left(s\right)}{\left\langle \Gamma_{\omega^{\prime },n^{\prime }}^{\left(s^{\prime }\right)}\left(\vec{\sigma }\right)\right\rangle }_{\omega ,n,s}$$

where the summation is performed over all the clusters, distinct under symmetry operations in the BCC lattice for the present study. $\vec{\sigma }$ denotes the ensemble of occupation variables in the lattice. ω and *n* are the cluster size and its shell label, respectively. $m_{\omega ,n}^{\left(s\right)}$ denotes the site multiplicity of the decorated clusters (in per‐lattice‐site units); and $J_{\omega ,n}^{\left(s\right)}$ represents the many‐body effective cluster interaction (ECI) energy corresponding to the same (*s*) decorated cluster. In Equation (1), ${\left\langle \Gamma_{\omega^{\prime },n^{\prime }}^{\left(s^{\prime }\right)}\left(\vec{\sigma }\right)\right\rangle }_{\omega ,n,s}$ denotes the cluster function, averaged over all the clusters of size, ω′, and label, *n*′, decorated by the sequence of point functions,(*s*′). Within the matrix formulation of CEH for a system with *K* elements, the cluster function is related to the probability function of finding cluster via the formula:^[^ ^103^ ^]^

(2) $$y_{\omega ,n}^{\left(AB\cdots \right)}={\overset{\omega }{\overset{︷}{\left({\bar{\bar{\tau }}}_{K}^{-1}⊗\cdots ⊗{\bar{\bar{\tau }}}_{K}^{-1}\right)}}}_{AB\cdots ,ij\cdots }\left\langle \Gamma_{\omega ,n}^{\left(ij\cdots \right)}\right\rangle$$

where the matrix elements of the inverse of the $\left({\bar{\bar{\tau }}}_{K}\right)$ matrix are defined by the expression:^[^ ^103^ ^]^

(3) $${\left({\bar{\bar{\tau }}}_{K}^{-1}\right)}_{ij}=\left\{\begin{array}{cc} \frac{1}{K} & \mathrm{if}j=0,\\ -\frac{2}{K}\cos\left(2\pi \left\lceil \frac{j}{2}\right\rceil \frac{\sigma_{i}}{K}\right) & \mathrm{if}j>0\mathrm{and}j-1<K\mathrm{and}j\mathrm{odd},\\ -\frac{2}{K}\sin\left(2\pi \left\lceil \frac{j}{2}\right\rceil \frac{\sigma_{i}}{K}\right) & \mathrm{if}j>0\mathrm{and}j\mathrm{even},\\ -\frac{1}{K}\cos\left(2\pi \left\lceil \frac{j}{2}\right\rceil \frac{\sigma_{i}}{K}\right) & \mathrm{if}j-1=K\mathrm{and}j\mathrm{odd}. \end{array}\right.$$

and *i*, *j* = 0, 1, 2, …(*K* − 1), *j* and $\left\lceil \frac{j}{2}\right\rceil$ stands for the ceiling function ‐ rounding up to the closest integer. From the Equation (2), the point probability function is written as:

(4) $$y_{1,1}^{A}=\sum_{s}{\left({\bar{\bar{\tau }}}_{K}^{-1}\right)}_{A,(s)}\left\langle \Gamma_{1,1}^{\left(s\right)}\right\rangle$$

and the pair probability function is determined by the following formula:

(5) $$y_{2,n}^{AB}=\sum_{s}{\left({\bar{\bar{\tau }}}_{K}^{-1}⊗{\bar{\bar{\tau }}}_{K}^{-1}\right)}_{A,B,(s)}\left\langle \Gamma_{2,n}^{\left(s\right)}\right\rangle$$

Equation (5) allows the two‐body cluster probability to be linked with the Warren‐Cowley short‐range order (SRO) parameter, $\alpha_{2,n}^{\left(AB\right)}$, via the definition:^[^ ^117^, ^118^ ^]^

(6) $$y_{2,n}^{AB}=x_{A}x_{B}\left(\;1-\alpha_{2,n}^{AB}\right)\;$$

where *x*_*A*_ and *x*_*B*_ denote the bulk concentration of the chemical species A and B, respectively. In the case where $\alpha_{\text{2,n}}^{\text{AB}}=0$, the pair probability was given by the product of their concentrations x_B_x_B_ corresponding to random configuration of A and B species in an alloy system. In the case of $\alpha_{\text{2,n}}^{\text{AB}}>0$, clustering or segregation between A‐A and B‐B pairs was favored and for $\alpha_{\text{2,n}}^{\text{AB}}<0$, the chemical ordering of A‐B pairs occurs. By combining Equations (4),(5) and (6), the SRO parameters for *K*‐component system can be calculated by the general expression as follows:^[^ ^102^ ^]^

(7) $$\alpha_{2,n}^{AB}=1-\frac{\sum_{s}{\left({\bar{\bar{\tau }}}_{K}^{-1}⊗{\bar{\bar{\tau }}}_{K}^{-1}\right)}_{A,B,(s)}\left\langle \Gamma_{2,n}^{\left(s\right)}\right\rangle }{\left(\sum_{s}{\left({\bar{\bar{\tau }}}_{K}^{-1}\right)}_{A,(s)}\left\langle \Gamma_{1,1}^{\left(s\right)}\right\rangle \right)\left(\sum_{s}{\left({\bar{\bar{\tau }}}_{K}^{-1}\right)}_{B,(s)}\left\langle \Gamma_{1,1}^{\left(s\right)}\right\rangle \right)}$$

The expression to calculate the average SRO parameter for first and second nearest neighbors in a BCC lattice is given by:^[^ ^119^ ^]^

(8) $$\alpha_{avg}^{AB}=\frac{8\alpha_{2,1}^{AB}+6\alpha_{2,2}^{AB}}{14}$$

where $\alpha_{2,1}^{AB}$ and $\alpha_{2,2}^{AB}$ are the first and second nearest neighbors SRO parameters, respectively.

The CEH, defined by Equation (1) can be used to explicitly determine the configuration entropy of a *K*‐component system via the thermodynamic integration method.^[^ ^115^, ^116^ ^]^ Here, the entropy was computed from fluctuations of the enthalpy of mixing at a given temperature using the following formula

(9) $$\begin{array}{c} S_{conf}\left[T\right]=\;\int_{0}^{T}\;\frac{C_{conf}\left(T^{\prime }\right)}{T^{\prime }}dT^{\prime }=\;\int_{0}^{T}\;\frac{\left\langle {\left[\Delta H_{mix}^{CEH}\left(T^{\prime }\right)\right]}^{2}\right\rangle \;-\;{\left\langle \left[\Delta H_{mix}^{CEH}\left(T^{\prime }\right)\right]\right\rangle }^{2}}{{T^{\prime }}^{3}}dT^{\prime } \end{array}$$

where $\left\langle {\left[\Delta H_{mix}^{CEH}\left(T^{\prime }\right)\right]}^{2}\right\rangle$ and ${\left\langle \left[\Delta H_{mix}^{CE}\left(T^{\prime }\right)\right]\right\rangle }^{2}$ are the square of the mean and mean square enthalpies of mixing, respectively. The statistical average over configurations at finite temperature in Equation (9) can be performed by combining the CEH with AMC technique.

The CEH was constructed by mapping a large database of DFT energies of not only randomized solution structures but also for intermetallic ordered structures with the different compositions.^[^ ^102^, ^104^, ^105^, ^106^ ^]^ Within the present study for W–Ta–V alloys, the set of ECIs energies obtained from the recent study^[^ ^105^ ^]^ had been employed as it took into consideration not only two and three but also four‐body cluster interaction which was crucial in discovering the important role of the CSRO V‐Ta CSRO parameter. In this work, semi‐canonical exchange AMC simulations were performed using Alloy Theoretic Automated toolkit (ATAT)^[^ ^120^, ^121^ ^]^ package for the BCC 20 × 20 × 20 super‐cell with 16000 atoms. For each composition of the ternary W–Ta–V alloys, the simulation computes the enthalpy of mixing at each temperature step for reaching a thermodynamic equilibrium configuration. The start temperature for each simulation begins at 2000 K and was reduced to 10 K with temperature steps of 10 K. The start temperature was chosen such that a disordered configuration was near‐guaranteed after 2000 MC steps per atom in the thermalization and accumulation stages. The free energy of an alloy at each temperature was calculated from the enthalpy of mixing and the configuration entropy determined by Equation (9). The error bars of CSRO parameters can be determined by Equation (7) using AMC simulations of average cluster functions calculated from thermodynamic equilibrium configurations generated for each temperature and alloy composition.

### Molecular Dynamics Simulations

The molecular dynamics simulations were performed with the LAMMPS package^[^ ^122^ ^]^ with an accurate machine‐learned (ML) interatomic potential (see tabGAP^[^ ^123^, ^124^ ^]^). The ML potential was trained to energies, forces, and virials of a diverse database of structures computed with DFT. The training structures span the full composition space of alloys and include defects and liquids to make the potential applicable to radiation damage simulations. The database builds on the one from Byggmastar et al.,^[^ ^123^ ^]^ but was more tailored to W–Ta–V alloys by removing all Nb‐ and Mo‐containing structures. Details on the training and validation of the new tabGAP would soon be published elsewhere.

Radiation damage accumulation was simulated by performing successive collision cascades with primary knock‐on energies of 10 keV given in random directions to an atom close to the center of the simulation cell. Each cascade was simulated for 20 ps in the *NVE* ensemble, with *NVT* along the simulation cell borders (8 Åthickness) to dissipate heat and dampen shock waves generated by the cascade. This was followed by 10 ps with all atoms in the *NPT* ensemble to stabilise the temperature and pressure. An adaptive time step and electronic stopping as a friction term with data from SRIM^[^ ^112^ ^]^ were used during the cascade phase. The temperature was 1073 K (same as in the experiments) and the pressure zero. After every cascade, the simulations cell was randomly shifted to achieve homogeneous irradiation. The size of the simulation cell was close to 20 nm in each dimension, equalling 498 960 atoms and roughly the size of one grain in the synthesized thin films. The atoms were randomly ordered with the same alloy compositions as in experiments. The dose in dpa was calculated with the NRT‐dpa equation^[^ ^125^ ^]^ using a threshold displacement energy of 40 eV, consistent with the experiments. A total of 2500 successive cascades in each material were reached, corresponding to 0.4 dpa. Defects (interstitials, vacancies, dislocations) were analyzed after every cascade, using Wigner‐Seitz analysis,^[^ ^126^ ^]^ and DXA.^[^ ^127^ ^]^

At 0.4 dpa, the three materials were annealed in MD at 2000 K for 10 ns in the *NPT* ensemble. The high temperature was chosen to activate significant defect migration and hence possible recombination even at the limited MD nanosecond time scale. The defects were analyzed in the same way as above and visualized using Ovito.^[^ ^128^ ^]^

## Conflict of Interest

The authors declare no conflict of interest.

## Author Contributions

Conceptualization and visualisation is performed by M.A.T. and O.E.A. Methodology, investigation, data curation, and formal analysis is performed by all authors. Resources are provided by all authors. Funding acquisition is provide by M.A.T., D.N.M., and O.E.A. Writing original draft is provided by M.A.T. Writing review and editing is done by all authors. Project administration is performed by O.E.A.

## Supporting information

Supporting Information

## Data Availability

The raw data required to reproduce these findings are available in the Mendeley Data permanent repository: https://data.mendeley.com/datasets/y326fsd8ww/1
