# Supplementary material for: High Radiation Resistance in the Binary W‐Ta System Through Small V Additions: A New Paradigm for Nuclear Fusion Materials
Source: Adv Sci (Weinh). 2025 Mar 7;12(20):2417659. doi: 10.1002/advs.202417659 (PMC12120728; doi:10.1002/advs.202417659)
Supplement: Supplementary file 1 — Supporting Information [file ADVS-12-2417659-s001.docx]

**Supplementary Information**

The Supplementary Information contains additional results obtained using a combination of density functional theory, the cluster expansion Hamiltonian (CEH) method and atomistic Monte Carlo (AMC) simulations, which are not necessary for the main part of the manuscript but might be useful for the readers. It includes the structures of voids in binary W_44_Ta_56_ alloy with vacancies, and the results for W_53_Ti_42_V_5_ alloy showing chemical short-range order parameters (average, 1NN, 2NN), atomic structures at 100K and 1000K, enthalpy of mixing, configuration entropy and free energy.

**S1. Voids in W_44_Ta_56_ alloy**

To understand the origin and behaviour of voids in the irradiated W_44_Ta_56_ alloy, shown in Figs. 4A and 4D in the main text, AMC simulations were performed using the CEH model from Ref. [1] for the W_44_Ta_56_ alloy with 0.5% vacancies. The simulation involved a 40x40x40 bcc supercell with 128000 atomic sites, equivalent to dimensions of 12.6 nm. The temperature for AMC simulations was set at 1100 K, corresponding to the temperature of irradiation in the experimental setting. As shown in Fig. S1(a), the W and Ta atoms form in the W_44_Ta_56_ alloy at 1100 K the disordered solid solution. The vacancies within the alloy exhibit an attractive interaction, leading to the formation of voids, as depicted in Fig. S1(b). The AMC simulation cell contains four voids with dimensions of 1.1 nm, 1.4 nm, 1.7 nm, and 1.9 nm. The average void size is calculated as 1.5 nm, with a standard deviation of 0.4 nm. This observation aligns with transmission electron microscopy findings for the W_44_Ta_56_ alloy irradiated at 1073 K, as presented in Figs. 4A and 4D in the main article and agrees with the average void size of 1.66 ± 0.02 nm reported in Table 2 of the main text.


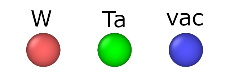


a)
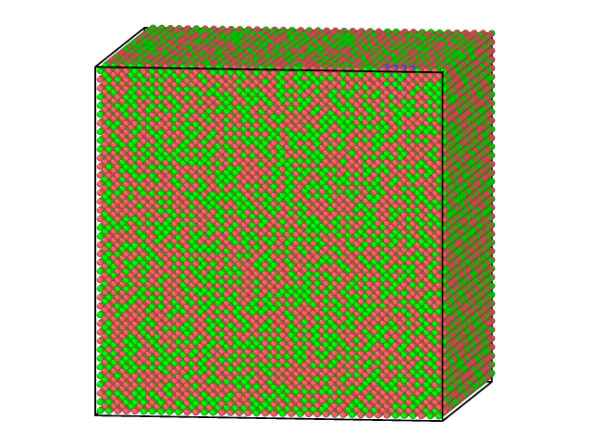
b)
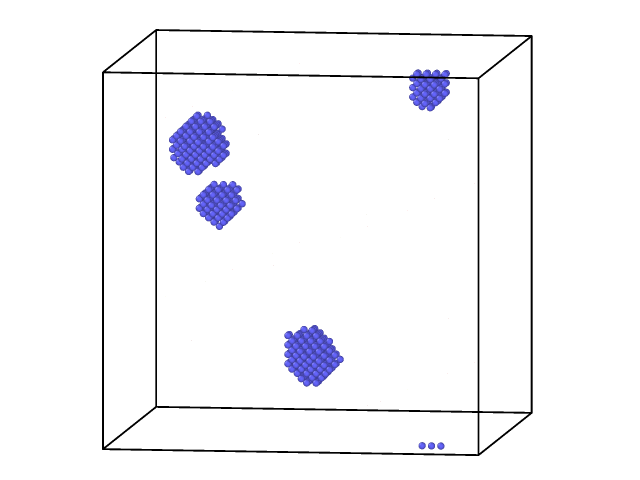


Figure S1: (a) The structure of W_44_Ta_56_ alloy obtained using DFT-based Monte Carlo simulations at 1100 K; (b) The same structure without showing the atoms of W and Ta.

**S2. Short-range ordering in W_53_Ti_42_V_5_ alloy**

To investigate the impact of substituting Ta atoms with Ti atoms in the W_53_Ta_42_V_5_ alloy, MC simulations were conducted using the CEH model from Ref. [2] for the W_53_Ti_42_V_5_ alloy. As shown in Fig. S2, the SRO parameter of Ti-V pairs in the W_53_Ti_42_V_5_ alloy look like the results obtained for Ta-V pairs the W_53_Ti_44_V_3_ alloy, shown in Fig. 5 in the main text. In both cases, the formation of V-W-rich phase is observed at 100 K, and the order-disorder transition temperatures are present at around 300 K.


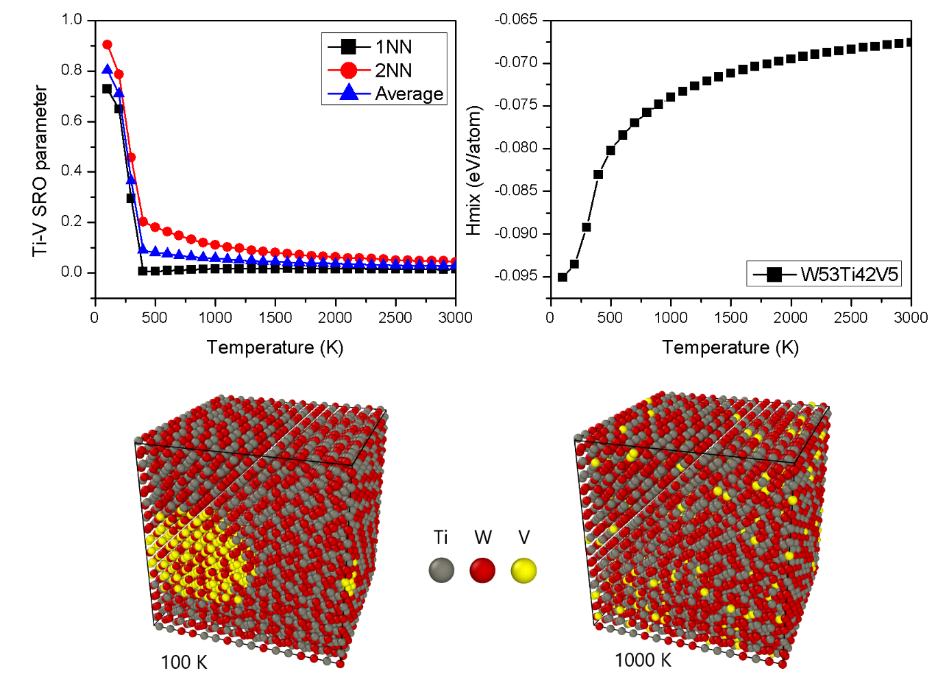


Figure S2: The Ti-V short-range order parameters for the first nearest-neighbour, second nearest-neighbour, the average values (top left), and the enthalpy of mixing (top right) obtained using DFT-based AMC simulations for W_53_Ti_42_V_5_ alloy as a function of temperature, as well as the representative structures of the alloy obtained from the simulations at 100 K (bottom left) and 1000 K (bottom right).

**S3. The presence of voided grain boundaries in the pristine W—Ta—V RHEAs**

Voided grain boundaries are observed in the pristine magnetron-sputtered material after deposition. This is illustrated in both bright-field (BF) and HAADF Scanning Transmission Electron Microscopy (STEM) micrographs in Figure S3. In BF-STEM, the voided grain boundaries are of light contrast while under HAADF, the voided grain boundaries are of dark appearance.


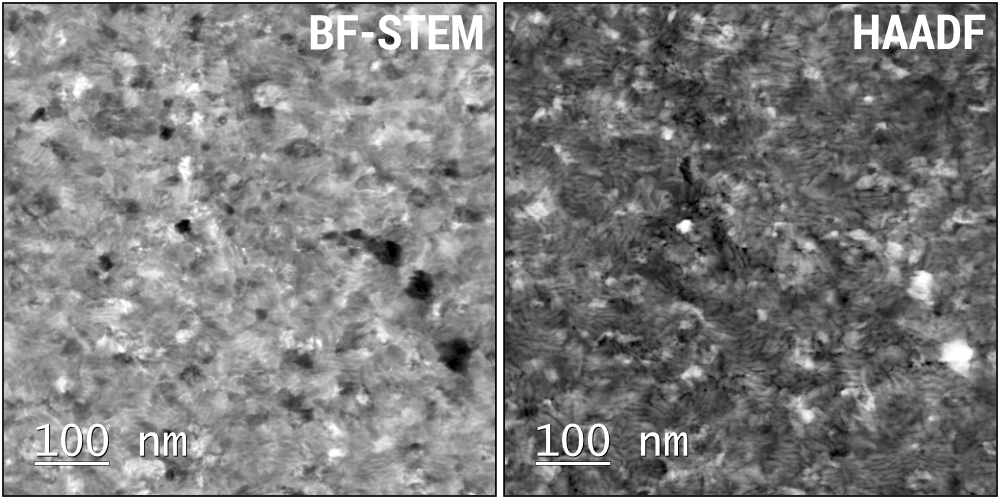


Figure S3: BF-STEM and HAADF micrographs points to the existence of voided grain boundaries in the pristine materials after magnetron-sputtering deposition.

**S4. STEM-EDX elemental maps of pristine and annealed conditions**

The elemental maps of the W_53_Ti_44_V_3_ alloy in both pristine and annealed conditions are shown in Figure S4. No segregation or secondary phases have been identified.

**
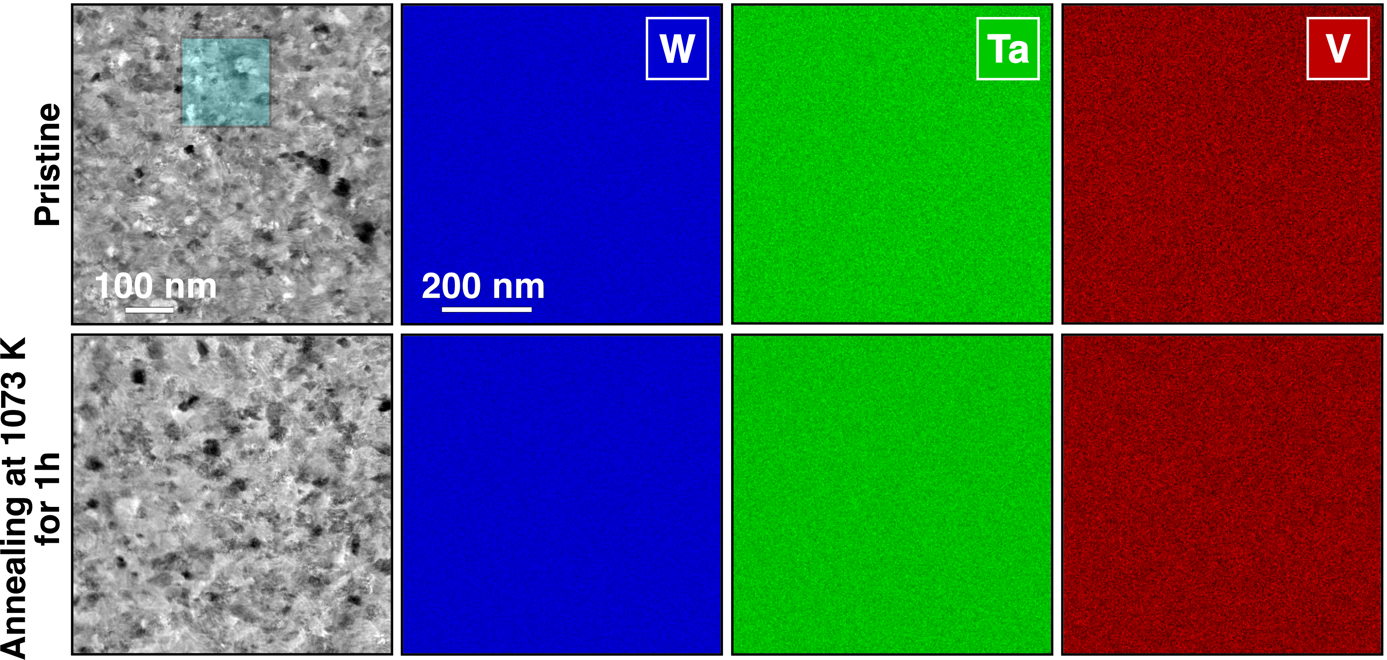
**

Figure S4: STEM-EDX elemental maps of the W_53_Ti_44_V_3_ alloy before irradiation and after annealing.

**S5. Irradiation damage profile calculated using SRIM2013-Pro**

**
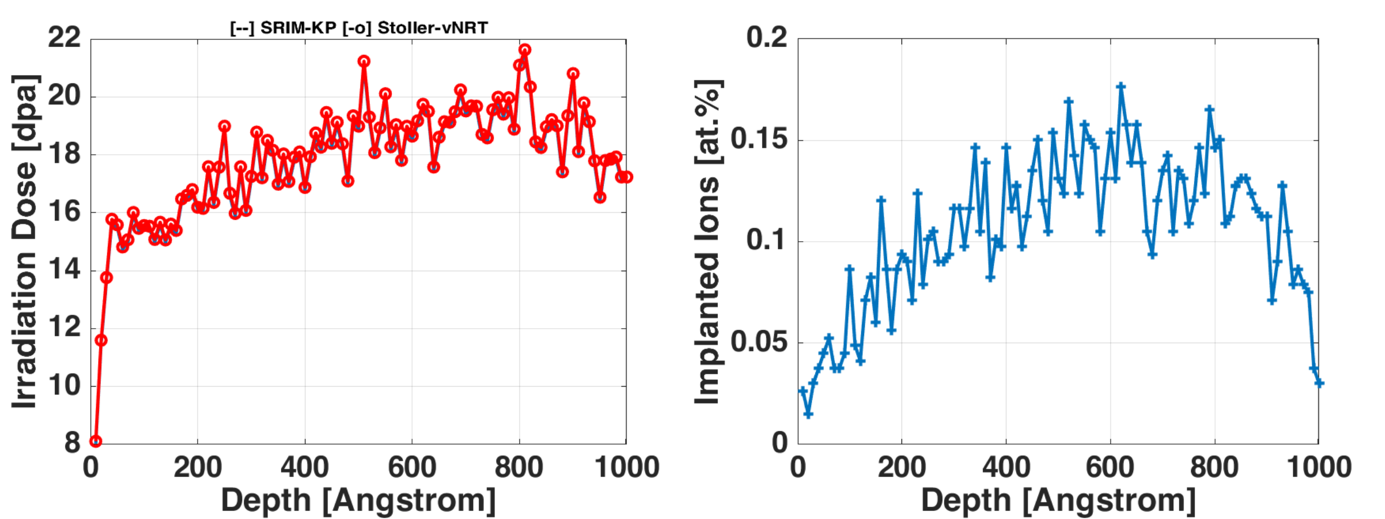
**

Figure S5: Irradiation dose as a function of depth (left) and implantation profile (right).

| **** |
| --- |
| Figure S6: Void diameters after irradiation up to 20 dpa at 1073 K. |

**S6. Experimental distribution of voids in all alloys studied**

The distributions of void sizes (diameter) for all the three alloys investigated in this work are shown in Figure S6. The lowest value is for the W_53_Ta_42_V_5_ alloy which is 1.19±0.01 nm. The void diameter for the W_52_Ta_44_V_3_ alloy was estimated to be 1.65±0.01 nm and for the binary W_44_Ta_56_ the void diameter was roughly 2.37±0.01 nm. Note: the error is the standard error of the mean.

**References**

[1] Nguyen-Manh, D. *et al.* First principles model for voids decorated by transmutation solutes: Short range order effects and application to neutron irradiated tungsten. *Physical Review Materials* 5, 065401 (2021).

[2] Sobieraj, D. *et al.* Chemical short-range order in derivative Cr–Ta–Ti–V–W high entropy alloysd from first-principles thermodynamic study. *Physical Chemistry and Chemical Physics* 22, 23929 (2020).
